# Can Family Planning Service Statistics Be Used to Track Population-Level Outcomes?

**DOI:** 10.9745/GHSP-D-17-00341

**Published:** 2018-03-21

**Authors:** Robert J Magnani, John Ross, Jessica Williamson, Michelle Weinberger

**Affiliations:** a Avenir Health, Glastonbury, CN, USA.; b Independent consultant, New Paltz, NY, USA.

## Abstract

The need for annual family planning program tracking data under the Family Planning 2020 (FP2020) initiative has contributed to renewed interest in family planning service statistics as a potential data source for annual estimates of the modern contraceptive prevalence rate (mCPR). We sought to assess (1) how well a set of commonly recorded data elements in routine service statistics systems could, with some fairly simple adjustments, track key population-level outcome indicators, and (2) whether some data elements performed better than others. We used data from 22 countries in Africa and Asia to analyze 3 data elements collected from service statistics: (1) number of contraceptive commodities distributed to clients, (2) number of family planning service visits, and (3) number of current contraceptive users. Data quality was assessed via analysis of mean square errors, using the United Nations Population Division World Contraceptive Use annual mCPR estimates as the “gold standard.” We also examined the magnitude of several components of measurement error: (1) variance, (2) level bias, and (3) slope (or trend) bias. Our results indicate modest levels of tracking error for data on commodities to clients (7%) and service visits (10%), and somewhat higher error rates for data on current users (19%). Variance and slope bias were relatively small for all data elements. Level bias was by far the largest contributor to tracking error. Paired comparisons of data elements in countries that collected at least 2 of the 3 data elements indicated a modest advantage of data on commodities to clients. None of the data elements considered was sufficiently accurate to be used to produce reliable stand-alone annual estimates of mCPR. However, the relatively low levels of variance and slope bias indicate that trends calculated from these 3 data elements can be productively used in conjunction with the Family Planning Estimation Tool (FPET) currently used to produce annual mCPR tracking estimates for FP2020.

## Abstract (precis)

Estimates of the modern contraceptive prevalence rate (mCPR), a population-level indicator, that are derived directly from family planning service statistics lack sufficient accuracy to serve as stand-alone substitutes for survey-based estimates. However, data on contraceptive commodities distributed to clients, family planning service visits, and current users tend to track *trends* in mCPR fairly accurately and, when combined with survey data in new tools, can be used to approximate the annual mCPR in the absence of annual surveys.

## INTRODUCTION

Until the late 1960s, family planning service statistics and vital statistics systems were, for all intents and purposes, the sole sources of data for tracking population trends and family planning program performance.^1^^,^^2^ Routine family planning program data have a number of strengths. Among these are that they (1) are collected in connection with service delivery and thus entail very limited additional data collection costs; (2) provide high geographic detail, even down to the service delivery point level; and (3) are available frequently—usually monthly, and potentially in real time. Routine program data also have weaknesses. These include that they (1) are prone to error (e.g., recording and processing errors, facility underreporting, duplicate reporting of clients who visit more than 1 service delivery point during a given reporting period, reporting delays, and deliberate padding of numbers) and (2) generally do not measure population-level indicators well, due in part to the above errors and in part to limited coverage of the contributions of the private sector (i.e., NGO and commercial providers of family planning).

In response to the limitations of service statistics data and vital statistics systems, and to the challenging and often lengthy processes required to reform them,^3^ a shift toward greater reliance on data from large-scale surveys was well underway by the early 1970s. This shift was led by global survey programs such as the World Fertility Survey (WFS) and the Contraceptive Prevalence Surveys (CPS) in the 1980s, followed by the Demographic and Health Surveys (DHS) and most recently the Performance Monitoring and Accountability 2020 (PMA2020) surveys.^4^ A number of countries also conduct frequent, multipurpose national surveys that collect relevant data. Although virtually all countries continue to collect and process family planning service statistics on a routine basis, most countries and the international family planning community at large tend to rely more heavily on data from large-scale surveys to track national and global family planning progress.

Virtually all countries collect and process family planning service statistics on a routine basis.

**FIGURE 1.**
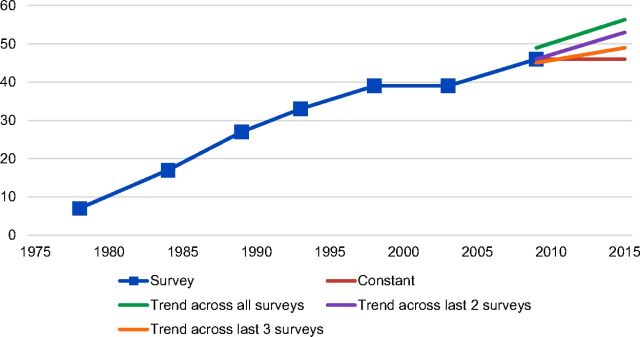
Options for Projecting the mCPR Trend Since the Last Large-Scale Survey Abbreviation: mCPR, modern contraceptive prevalence rate.

Recent years have, however, witnessed a renewed interest in family planning service statistics (and program data more generally). One reason is that the global Family Planning 2020 (FP2020) initiative requests that countries provide updates on a number of FP2020's Core Indicators on an annual basis. This leaves countries that rely on large-scale surveys, which are generally undertaken only every 3 to 5 years, in a difficult situation. In the absence of annual survey measurements, countries must project values of key indicators for each year since the last large-scale survey, pending information from the next large-scale survey (see Figure 1 for a visual depiction of the problem).

A second reason for the current interest in service statistics and program data, not related to FP2020, is that the high cost of national surveys has prompted some countries and international organizations to question whether reliance on surveys is cost-effective over the medium to long term. Discussions on this issue are by no means limited to family planning; for example, similar discussions about reducing the need for large-scale surveys are ongoing with regard to HIV and AIDS program data.^5^

Because trustworthy health information systems are essential to effective public health program management,^5^ interest in improving routine data for family planning and other program areas has spawned numerous efforts to develop new tools and processes. Examples include the Performance of Routine Information System Management (PRISM)^6^ and District Health Information System 2 (DHIS 2)^7^ initiatives. Such tools are especially needed to reform government health information systems in low- and middle-income countries, and many countries have indeed shifted to electronic health management information systems (HMIS) in recent years. This shift has created new opportunities for greater use of service statistics. So far, however, the impact of these efforts on improving routine data system functioning for family planning or other health programs has not been systematically documented.

Given the ongoing need for credible, annual family planning program tracking data for FP2020, we sought to assess the extent to which a set of commonly recorded and reported data elements in routine service statistics systems could, with some fairly simple adjustments, track key population-level outcome indicators. Two main questions were addressed in this study: (1) How well do approximations of the modern contraceptive prevalence rate (mCPR) derived from service statistics track survey-based mCPR estimates? (2) Do some data elements perform better than others such that they should be preferred for tracking purposes? There is at present little hard evidence available on these questions, yet the answers have important implications for how low- and middle-income countries can go about tracking national family planning program performance in the short term while information system development efforts proceed.

The Family Planning 2020 initiative requires ongoing family planning performance data.

## METHODS

We collected service statistics data from 22 FP2020 pledging countries that are being supported by the Track20 Project. The countries included in the analyses are listed in Supplement 1. We considered 3 family planning data elements: (1) number of contraceptive commodities distributed to clients, by method; (2) number of client family planning service visits, by method; and (3) number of current contraceptive users. (See the Box for more detailed definitions of these elements.) Data used were from government databases and reports in the respective countries. To increase the robustness of the results, we required each country included in the analyses to have at least 3 years of data for at least 1 of the 3 data elements, including at least 1 year of data that overlapped with a large-scale survey. The service statistics data elements we analyzed are summarized in Table 1, together with the number of countries that could supply each type of data.

**TABLE 1.** Countries Included in the Analyses, by Availability of Service Statistics Data Elements

Countries With Service Statistics Available (N=22)	Years of Data Available		
	3–4	5–6	≥7
**Commodities data only (n=10)**	4	1	5
**Commodities and visits data (n=4)**			
Commodities	2	1	1
Visits	1	2	1
**Commodities and users data (n=5)**			
Commodities		2	3
Users	1	3	1
**Visits and users data (n=1)**			
Visits			1
Users			1
**Commodities, visits, and users data (n=2)**			
Commodities		2	
Visits		2	
Users		1	1

BOX **Description of Family Planning Data Elements Used in the Study**

**Commodities distributed to clients:** The number of contraceptive commodities distributed to clients, such as the number of pill cycles and number of intrauterine devices (IUDs). Because our intent in using data on commodities to clients was to estimate the annual number of contraceptive users, we also included numbers of female and male sterilization services provided, although they do not involve commodities. We used data on commodities distributed from service delivery points—that is, counted when products or services are provided to clients—as opposed to further back in the supply chain, such as when products are distributed to warehouses or service delivery points.

**Service visits:** The number of times clients interacted with a provider for contraceptive services. For short-acting contraceptive methods, the same client may be counted multiple times because the client comes multiple times for resupply (e.g., an injectables client has 4 service visits because she receives 4 injections over the course of a year). The conversion of service visits data to an estimate of the number of contraceptive users in a given year must take this into account.

**Current users:** Persons who are currently using contraception, regardless of when the method was received. This is not directly comparable with the number of clients served in a year, because it includes people still using long-acting or permanent methods received in previous years (e.g., a woman who had an IUD inserted in 2012 may still be an IUD user in 2015). In service statistics systems, the estimated number of current users is calculated in one of several ways. Some countries calculate the number of current users for a given method in a year as the number of new users of the method plus the number of continuing or repeat users minus the number of dropouts or discontinuers. The challenges in producing the estimate are (1) to avoid double-counting clients and (2) to accurately track client dropouts or discontinuation.

The study compared service statistics data with the annual estimates of mCPR provided in the United Nations Population Division World Contraceptive Use dataset.^8^ The World Contraceptive Use (WCU) estimates were calculated using the Family Planning Estimation Model (FPEM) developed by the United Nations Population Division.^9^ FPEM is a Bayesian hierarchical model that fits curves to historical data. The model fits a logistic growth curve to the contraceptive prevalence rate (CPR) data from all available surveys to determine the long-term trend in contraceptive use. It uses a time-series model with autocorrelation to capture country-specific deviations around the long-term trend. The long-term trend moves toward an asymptote (where the *trend* levels off) with the *pace* and *timing* of the increase in contraceptive use determining the exact shape of the logistic curve. These 3 parameters—trend, pace, and timing—are estimated from national data and informed by regional and global trends and patterns. A second model splits total contraceptive use into modern and traditional methods. A third model fits trends in unmet need. Related outcomes such as total demand for family planning are then calculated. FPEM not only determines the most likely trends in family planning outcomes, but also estimates an uncertainty range around the trends so that each estimate contains a median estimate as well as a 95% uncertainty range. In fitting the models, FPEM distinguishes between different types of data (e.g., DHS versus other national surveys) and automatically assigns higher credibility to sources of data with a lower estimated error variance. (In the model, DHS is estimated to have the lowest error variance.)

We compared family planning service statistics data with World Contraceptive Use data.

To make comparisons with World Contraceptive Use estimates of mCPR,^8^ we first had to convert the service statistics to approximations of the mCPR. This was accomplished using a tool developed by Track20 called the Service Statistics to Estimated Modern Use (SS to EMU) tool.^10^ In the case of numbers of contraceptive commodities distributed to clients (by method) and numbers of client family planning service visits (by method), we converted annual counts into annual estimates of numbers of current or active contraceptive users. This was done differently for short-acting methods versus long-acting reversible contraceptives.

For short-acting methods, we estimated the number of users based on coverage needed for 1 year of contraceptive protection and estimated commodities to clients by applying couple-years of protection (CYP) conversion factors. Our estimate for service visits data was based on the average number of service visits needed per year to produce 1 CYP. Due to data limitations, it was not possible to account for the fact that not all client service visits are associated with new contraceptive use; for example, some consultations concern side effects with method use. As a result, estimates of total CYP based upon service visits data would tend to be biased upward. The conversion factors we used are documented in Supplement 2.

We used a more refined calculation for long-acting reversible contraceptives to account for continued use of intrauterine devices (IUDs) and implants from insertions in past years; the detailed calculations can be found in Supplement 3. As the purpose of the exercise was to estimate total users, counts of persons sterilized were included in the estimate based on commodities to clients data (these users are already included in the service visits data).

Finally, we introduced a correction to recognize that government statistics on commodities distributed to clients and on family planning service visits account for differing shares of the overall market for each method across countries. This is because the extent to which private-sector family planning service outputs are included in government statistics varies considerably by country. In many countries, some portions of the private sector (usually NGOs) report into the government HMIS such that their outputs are already represented in government service statistics. To compensate for this, we adjusted the underlying data elements (e.g., number of commodities to clients or number of client visits) upward by a quantity equivalent to the estimated private-sector market share for each particular method. The preferred source of data for calculating the correction factors was DHS data on where women access each contraceptive method in the respective countries.

The estimated numbers of contraceptive users, calculated as described above, were then divided by the estimated number of women of reproductive age in each country during each year covered by the service statistics data. Estimates and projections of the number of women of reproductive age from the United Nations Population Division were used as denominators in the calculations.^11^ This yielded approximations of annual mCPR estimates, referred to as estimated modern use (EMU) rates. EMU rates constitute an approximation of the actual mCPR, and as such we retain the label EMU rather than mCPR—to reinforce the point that they are approximations.

For the third type of service statistic, number of current contraceptive users, we used the absolute numbers provided by each country as numerators to calculate annual EMU rates, with the United Nations Population Division population data again providing the denominators for the calculations. The private-sector adjustment described above was also applied to the current users data.

The performance of the 3 data elements in tracking mCPR was assessed on the basis of their root mean square errors (RMSEs).^12^ RMSE is a commonly used measure of the accuracy of a given estimate or set of estimates in relation to the “true” value. In the present study, the RMSE may be thought of as the average difference between the service statistics–based estimates (EMUs) and the survey-based estimates of mCPR, over the period of time for which both service statistics–based and survey-based estimates are available. While acknowledging that the World Contraceptive Use estimates are not free of error, we used these estimates as the “gold standard” for measuring the accuracy of the mCPR approximations derived from service statistics.

We assessed the performance of 3 types of program data in tracking mCPR.

Total mean square errors (MSEs), a measure of total measurement error, were calculated as the average of the squared differences of the EMUs (the service statistics–based estimates of mCPR) versus the survey-based mCPR estimates. That is:

*MSE* = ∑*(EMU_year i_* − *mCPR_WCU year i_) ^2^/n*

Where:

*EMU_year i_* = *the SS to EMU tool estimate of the mCPR for year i;*
*mCPR_WCU year i_* = *the corresponding World Contraceptive Use survey-based estimate of the mCPR for year i; and*
*n* = *the number of years for which service statistics–based estimates are available.*

The RMSE is simply the square root of the MSE. It is calculated in order to convert the result to the same metric as the input data (i.e., percentages rather than squared percentages). An RMSE of zero indicates perfect accuracy (which is of course unachievable in actual practice), and the level of total measurement error is indicated by the magnitude of the estimated RSME.

To further explore the sources of error in the service statistics data elements, we attempted to determine the magnitude of several types of errors. Noting that the MSE consists of both random and systematic error (in simple terms, MSE = Variance + Bias^2^), we estimated 3 error components:

- Random annual error (or variance)
- Systematic error or bias with regard to the level of the service statistics–based estimates
- Systematic error (bias) with regard to trend

**FIGURE 2.**
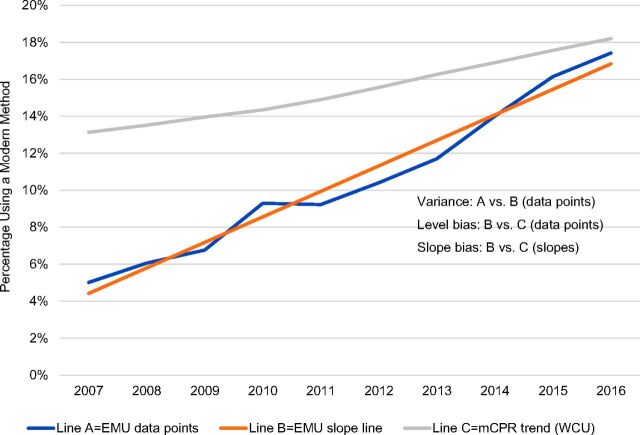
Variance, Level Bias, and Slope Bias as Components of Mean Square Error Abbreviations: EMU, estimated modern use; mCPR, modern contraceptive prevalence rate; WCU, World Contraceptive Use.

*Variance* consists of random measurement errors that do not affect the mean or expected value of the estimate(s). Variance can be thought of as random “noise” in the data. Such variability can be caused by actual annual fluctuations in service volume as well as by inconsistent recording and reporting of family planning service data from year to year. In the present application, we defined variance in terms of annual fluctuations about the least-squares linear trend line of EMU values for each of the 3 data elements (these amount to smoothed trend lines). Figure 2 presents a visualization using commodities to clients data from an unnamed country: Variance is calculated by comparing each of the annual EMU data points on line A (the blue line) against the respective annual values on the EMU trend or slope line represented by line B (the orange line). Although trends in EMU are not necessarily linear, any departure from linearity is unlikely to seriously distort the study findings over the relatively short intervals of time for which service statistics are available (see Table 1). Because countries with higher EMU rates have the potential for greater variability in EMU estimates in absolute terms, compared with countries with lower EMU rates, we used relative variance (RelVar), which expresses variability as a ratio to the level of the statistic being measured (EMU rates in the present case).

*Bias*, unlike variance, pertains to systematic measurement error; that is, errors that alter the mean or expected value of the estimate(s). Bias can be caused by consistent underreporting or overreporting of numbers of commodities distributed to clients, service visits, or current contraceptive users; by service delivery points not reporting data; and by a lack of private-sector data resulting in underreporting of services being provided nationally.

Two measures of bias were calculated in the analyses. *Level bias* was defined as the mean difference (absolute values) in level between the least-squares trend line of EMU estimates and the annual survey-based mCPR estimates. In Figure 2, this is represented by comparing the respective data points on the EMU trend or slope line (line B) against the corresponding annual estimates on the survey slope or trend line (line C, the gray line). This provides a measure of the extent to which the EMU rates are systematically higher or lower than the survey-based estimates of mCPR, and the magnitude of such systematic differences. As with estimates of variance, we expressed level bias relative to the mean of the survey-based mCPR estimates during the relevant time interval to account for the fact that countries with higher mCPRs had potentially higher levels of bias measured in absolute terms.

Error in estimates based on program data was measured via variance and 2 measures of bias.

*Slope bias* was defined as the absolute value of the difference between 2 slopes: the EMU least-squares slope or trend line (line B in Figure 2) versus the least-squares slope or trend line for the survey-based estimates of mCPR (line C in Figure 2). Slope bias quantifies how well the trend in approximate mCPR derived from service statistics (i.e., the EMU rate) tracks the trend in survey-based estimates of mCPR. It is possible for estimates based on service statistics to track the trend in survey-based estimates reasonably well despite variance and level bias, and we used the measure of slope bias to assess the extent to which this was the case in the countries included in the analyses. It can be empirically demonstrated that the absolute value of the differences between slopes accurately captures differences irrespective of whether the slope coefficients have different signs (one positive, one negative) or merely differences in magnitude (with the same sign). In Figure 2, slope bias may be visualized as the difference in slope or trend between lines B and C.

Computational details for the components of MSE may be found in Supplement 4.

## RESULTS

MSE calculations for all countries (unnamed) are documented in Supplement 5. The data shown in Supplement 5 were extracted and organized in different ways for presentation purposes. Table 2 shows data from all countries that met the inclusion criteria outlined in the Methods section. The numbers of countries on which the results for each component are based are shown at the top of the respective columns of the table; figures show median values. The first row shows the RMSEs for the 3 family planning service statistics considered in the study, while the bottom 3 rows show the median figures for variance, level bias, and slope bias errors, respectively.

**TABLE 2.** Median MSE Results Across Countries Providing at Least 1 Service Statistics Data Element

MSE Component	Commodities to Clients Data (21 Countries)	Service Visits Data (7 Countries)	Current Users Data (8 Countries)
RMSE	0.0695	0.0961	0.1864
Variance	0.0014	0.0002	0.0012
Level bias	0.3114	0.3452	0.5774
Slope bias	0.0114	0.0069	0.0104

Abbreviations: MSE, mean square error; RMSE, root mean square error.

RMSEs were modest for all commodities distributed to clients and service visits data (7% and 10%, respectively), but somewhat higher for current contraceptive users data (19%). Variance and slope bias tended to be relatively small for all 3 data elements. Level bias, on the other hand, was far and away the largest contributor to tracking error. Median levels of level bias ranged from a low of 31% for commodities data to a high of 58% for current users data, even with the several adjustments made in our analyses. Estimates of mCPR derived from service statistics (e.g., EMUs) tended to be lower than the estimates derived from surveys, but this tendency was far from universal (Table 3). Estimates based upon current users data were in fact equally likely to be higher versus lower than the corresponding survey-based estimates.

**TABLE 3.** Relationship Between Service Statistics Estimates and Survey-Based Estimates of mCPR

Relationship	Commodities to Clients Data (21 Countries)	Service Visits Data (7 Countries)	Current Users Data (8 Countries)
Service statistics estimates always < survey estimates	14	5	3
Service statistics estimates always > survey estimates	5	1	4
Varies by year	2	1	1

Abbreviation: mCPR, modern contraceptive prevalence rate.

Level bias was the largest contributor to error in program data estimates.

Because different sets of countries contributed to the figures for the respective data elements shown in Table 2, there is the danger that the comparisons shown in the table are confounded by country differences in the underlying quality of service statistics, irrespective of type of data. If this were to be the case, the data shown in Table 2 would be less than optimal for addressing the question of which of the 3 service statistics data elements performs best in tracking mCPR. To draw more valid conclusions, we undertook a series of paired comparisons among countries that could provide 2 or more of the data elements. The results, shown in Table 4, indicate an advantage for commodities to clients data versus both service visits and current users data with regard to overall RMSE. The advantage was somewhat larger when comparing commodities to clients data with current users data, but we noted that the slope bias for the users data was slightly lower than for the commodities data. The results for service visits versus current users data were based upon too few countries to draw firm conclusions, but the data available suggested no clear preference.

**TABLE 4.** Pairwise Comparisons of Median MSE Across Countries Providing at Least 2 Service Statistics Data Elements

MSE Component	Commodities vs. Visits (6 Countries)		Commodities vs. Users (7 Countries)		Visits vs. Users (3 Countries)	
	Commodities Data	Visits Data	Commodities Data	Users Data	Visits Data	Users Data
RMSE	0.0859	0.1182	0.1019	0.2216	0.2163	0.2115
Variance	0.0009	0.0001	0.0010	0.0013	0.0003	0.0008
Level bias	0.4054	0.4483	0.3103	0.5664	0.6015	0.5904
Slope bias	0.0029	0.0112	0.0161	0.0079	0.0158	0.0129

Abbreviations: MSE, mean square error; RMSE, root mean square error.

Note: Data from the 2 countries that collect all 3 types of service statistics are included in this table.

## DISCUSSION

The study findings are instructive with regard to the potential for making greater use of service statistics to track the progress of national family planning programs in low- and middle-income countries. On the less positive side, RMSEs ranged from 7% to 19%, and the results suggest that despite improvements in national health information systems used for family planning service statistics and the analytic adjustments introduced in this study, none of the family planning data elements we addressed tend to track survey-based estimates of mCPR very well with regard to level. Of the components of MSE, level bias was consistently the largest, and by a considerable margin. It is possible that making an additional adjustment for non-reporting by service delivery points would further reduce level bias, but our experience with some countries during related work indicates that this will not always be the case.^13^^,^^14^ We did not attempt such adjustments in the analyses reported here due to insufficient consistency in the reporting rate information provided by the participating countries.

Estimates based on family planning program data alone did not match mCPR estimates based on survey data.

On a more encouraging note, our results indicate that variance errors—or annual fluctuations in service volumes—tend not to be sufficiently large as to compromise annual tracking. This is important, as even with low levels of bias, annual estimates with high variability from year to year would not be of much practical use in tracking program performance. Yet more encouraging is that the slopes or trend lines constructed from the service statistics tend to track the trend lines constructed from survey data reasonably well.

In view of these findings, **how might one best use family planning service statistics to track family planning program performance at the population level?** Our recommended approach is not to use service statistics data to make direct, stand-alone estimates of CPR or mCPR, but rather to use them in conjunction with the Family Planning Estimation Tool (FPET).^15^ FPET is a modified version of the United Nations Population Division's FPEM that is designed to (1) permit individual countries to access the model to run country-specific analyses and projections and (2) incorporate service statistics into the estimation process. Individual countries can use these capabilities to obtain “best estimates” of past trends and to project them to years subsequent to the most recent national survey. A limitation of FPET is that the uncertainty bounds around annual estimates of mCPR grow large very quickly as the number of years since the last survey increases. On the other hand, a benefit of FPET is that including service statistics data informs only the trend (slope) of the estimates, thus avoiding the level bias observed in this study (the level of FPET estimates are informed by survey-based data, which are assumed to be more accurate). The key point is that the combination of the 2 sources—service statistics and FPET—employs the information from each that it is best positioned to provide. Further information on FPET, including instructions for using service statistics along with survey data, may be found on the Track20 Project website.^15^ Note that 13 countries incorporated service statistics into their FPET projections for the 2017 FP2020 annual report.^16^

Service statistics data can be used in conjunction with the Family Planning Estimation Tool to produce better estimates of family planning program performance at the population level than using service statistics data alone.

**Is there a preferred data element for tracking program progress at the population level?** Our analyses, albeit based upon a limited number of countries in which it was possible to make country-specific, paired comparisons, indicate a preference for commodities to clients data, but with the important nuance that none of the 3 data elements we considered performs well in tracking *levels* of mCPR. However, all 3 data elements appear to perform well enough with regard to tracking mCPR *trends* to be useful in FPET applications as described above. The individual country calculations provided in Supplement 5 demonstrate considerable variability from country to country. In view of this, the choice of preferred data element is best made on a country-by-country basis, depending upon the relative performance of the respective data elements in each country. More countries collect data on commodities distributed to clients than data on client service visits or current contraceptive users, perhaps reflecting greater perceived utility, and in a sizable number of countries, commodities to clients data may be the only option available.

### Strengths and Limitations

This study is based on data from 22 countries spanning both Asia and Africa. Population-based surveys make results and underlying datasets publicly available; however, there is no global repository for or system for disseminating service statistics. This article represents one of the most comprehensive assessments of family planning service statistics available. While we feel that the wide selection of countries provides a robust overview, we recognize that our data reflect only a snapshot of health information systems across the developing world and that our conclusions may not fit all countries.

The countries included in our analyses self-selected to be pledging countries for the global FP2020 initiative and thus may be viewed as having above-average levels of government commitment to family planning among other low- and middle-income countries. Whether this translates into comparable commitment to and success in strengthening their routine data systems is unknown. We lack a comparable indicator of government commitment to improving routine data systems, and thus are unable to judge whether the countries included in the study are atypical in this regard.

Finally, we note that while countries are increasingly interested in making use of service statistics to generate estimates of population-level outcomes such as mCPR, for reasons described earlier in the article, service statistics data have merit and utility for other important purposes over and above their ability to track population-level outcomes. Among these purposes are routine program monitoring, microplanning at the facility level, and strategy development and decision making at the national and subnational levels.

## CONCLUSION

Estimates of the mCPR derived directly from family planning service statistics lack sufficient accuracy to serve as stand-alone substitutes for survey-based estimates. However, data on contraceptive commodities distributed to clients, and to a lesser extent family planning service visits and current users, have relatively modest variability from year to year and tend to track trends in mCPR fairly accurately. When used in conjunction with survey data and new estimation tools, they can be used to produce defensible annual approximations of the mCPR in the absence of annual surveys.

## Supplementary Material

17-00341-Magnani-Supplements.pdf

## Notes

**Peer Reviewed**

**First Published Online:** February 21, 2018

**Funding:** The Bill & Melinda Gates Foundation via a grant to Avenir Health.

## References

[1] Ross JA Watson WB Lapham RJ. Handbook for Service Statistics in Family Planning Programs. 3rd ed. New York: Population Council; 1971.

[2] Cuca R. Evaluation of family planning programs using service statistics. http://documents.worldbank.org/curated/en/405091468739227905/pdf/multi0page.pdf. International Bank for Reconstruction and Development, Economic Staff Working Paper No. 137. Prepared November 1972. Accessed January 18, 2018.

[3] Aqil A Lippeveld T Hozumi D. PRISM framework: a paradigm shift for designing, strengthening and evaluating routine health information systems. Health Policy Plan. 2009;24(3):217–228. 10.1093/heapol/czp010. 19304786 PMC2670976

[4] Bill & Melinda Gates Institute for Population and Reproductive Health. Performance Monitoring and Accountability 2020 (PMA2020) website. https://www.pma2020.org. Accessed January 24, 2018.

[5] AbouZahr C Boerma T. Health information systems: the foundations of public health. Bull World Health Organ. 2005;83(8):578–583. 16184276 PMC2626318

[6] MEASURE Evaluation. Tools for Data Demand and Use in the Health Sector: Performance of Routine Information Systems Management (PRISM) Tools. Chapel Hill, NC: Carolina Population Center, University of North Carolina-Chapel Hill; 2011. https://www.measureevaluation.org/resources/publications/ms-11-46. Accessed January 18, 2018.

[7] Health Information Systems Programme (HISP). DHIS 2 website. https://www.dhis2.org/. Accessed January 18, 2018.

[8] United Nations Department of Economic and Social Affairs, Population Division. World Contraceptive Use 2016. New York: United Nations Population Division; 2016. http://www.un.org/en/development/desa/population/publications/dataset/contraception/wcu2016.shtml. Accessed January 18, 2018.

[9] Alkema L Kantorova V Menozzi C Biddlecom A. National, regional, and global rates and trends in contraceptive prevalence and unmet need for family planning between 1990 and 2015: a systematic and comprehensive analysis. Lancet. 2013;381(9878):1642–1652. 10.1016/S0140-6736(12)62204-1. 23489750

[10] SS to EMU Tool. Track20 website. http://www.track20.org/pages/our_work/innovative_tools/SS_to_EMU_tool.php. Accessed January 18, 2018.

[11] United Nations Department of Economic and Social Affairs, Population Division. The World Population Prospects: 2015 Revision. New York: United Nations Population Division; 2015. http://www.un.org/en/development/desa/publications/world-population-prospects-2015-revision.html. Accessed January 18, 2018.

[12] Wackerly D Mendenhall W Scheaffer RL. Mathematical Statistics with Applications. 7th ed. Belmont, CA: Thomson Higher Education; 2008.

[13] Universitas Gadjah Mada Center for Health Policy and Management. Tracking mCPR from Service Statistics: A Methodological Study in Indonesia. Bulaksumur, Yogyakarta, Indonesia: Universitas Gadjah Mada; 2016. https://desentralisasi-me-kb.net/tracking-mcpr-from-service-statistics-a-methodological-study-in-indonesia/. Accessed January 18, 2018.

[14] Universitas Gadjah Mada Center for Health Policy and Management. Correcting for Non-Reporting in Routine Family Planning Program Data: Case Study in Four Indonesian Provinces. Bulaksumur, Yogyakarta, Indonesia: Universitas Gadjah Mada; 2016. https://desentralisasi-me-kb.net/correcting-for-non-reporting-in-routine-family-planning-program-data-case-study-in-four-indonesian-provinces/. Accessed January 18, 2018.

[15] New JR Alkema L. Family Planning Estimation Tool (FPET). Track 20 website. http://fpet.track20.org/fpet/. 2015. Accessed January 18, 2018.

[16] Family Planning 2020 (FP2020). The Way Ahead, 2016–2017. Washington, DC: FP2020; 2017. http://progress.familyplanning2020.org/en. Accessed January 30, 2018.

[17] Weinberger MB Fry K Boler T Hopkins K. Estimating the contribution of a service delivery organisation to the national modern contraceptive prevalence rate: Marie Stopes International's Impact 2 model. BMC Public Health. 2013;13(suppl 2):S5. 10.1186/1471-2458-13-S2-S5. 23902699 PMC3684538

