# Supplementary material for: Can Family Planning Service Statistics Be Used to Track Population-Level Outcomes?
Source: Glob Health Sci Pract. 2018 Mar 21;6(1):93–102. doi: 10.9745/GHSP-D-17-00341 (PMC5878083; doi:10.9745/GHSP-D-17-00341)
Supplement: 17-00341-Magnani-Supplements.pdf [file 17-00341-Magnani-Supplements.pdf]

Magnani RJ, Ross J, Williamson J, Weinberger M. Can family planning statistics be used to track population-level outcomes? *Glob Health Sci Pract*. 2018;6(1). https://doi.org/10.9745/GHSP-D-17-00341

**SUPPLEMENT 1.** List of Countries Included in the Analyses

Afghanistan

Benin

Burkina Faso

Cameroon

Côte d'Ivoire

Democratic Republic of the Congo

Guinea

India

Indonesia

Kenya

Malawi

Mauritania

Nepal

Niger

Pakistan

Rwanda

Sierra Leone

South Africa

Togo

Uganda

Vietnam

Zimbabwe

Magnani RJ, Ross J, Williamson J, Weinberger M. Can family planning statistics be used to track population-level outcomes? *Glob Health Sci Pract*. 2018;6(1). https://doi.org/10.9745/GHSP-D-17-00341

**SUPPLEMENT 2.** Data Conversion Factors

## A. Commodities Distributed to Clients

| METHOD | | CYP FACTOR | UNITS |
|---|---|---|---|
| **Long Acting and Permanent Methods** | | | |
| Sterilization | Tubal Ligation (F) | 10 | years of protection |
| | Vasectomy (M) | 10 | years of protection |
| IUD | Copper- T 380-A IUD | *Alternative calculation used* | |
| | LNG-IUS | | |
| Implant | Implanon | | |
| | Jadelle | | |
| | Sino-Implant | | |
| **Short-Term Methods** | | | |
| Injectable | Depo Provera (DMPA) | 4 | per user per year |
| | Noristerat (NET-En) | 6 | per user per year |
| | Lunelle | 13 | per user per year |
| | Sayana Press | 4 | per user per year |
| | Other Injectable | 4 | per user per year |
| Pill | Standard daily Pill | 15 | per user per year |
| | Progestin only Pill | 15 | per user per year |
| | Peri-coital contraceptio | 15 | per user per year |
| Condom | Male Condom | 120 | per user per year |
| | Female Condom | 120 | per user per year |
| Other | LAM | 4 | per user per year |
| | SDM (Standard Days) | 1.5 | years of protection |
| | Vaginal barrier | 1 | per user per year |
| | Spermicides | 120 | per user per year |
| Emergency contraception | EC | 20 | per user per year |

## B. Service Visits

| METHOD | | VISIT FACTOR | UNITS |
|---|---|---|---|
| **Long Acting and Permanent Methods** | | | |
| Sterilization | Tubal Ligation (F) | 10 | years of protection |
| | Vasectomy (M) | 10 | years of protection |
| IUD | Copper- T 380-A IUD | *Alternative calculation used* | |
| | LNG-IUS | | |
| Implant | Implanon | | |
| | Jadelle | | |
| | Sino-Implant | | |
| **Short-Term Methods** | | | |
| Injectable | Depo Provera (DMPA) | 4 | visits per year |
| | Noristerat (NET-En) | 6 | visits per year |
| | Lunelle | 13 | visits per year |
| | Sayana Press | 4 | visits per year |
| | Other Injectable | 4 | visits per year |
| Pill | Standard daily Pill | 4 | visits per year |
| | Progestin only Pill | 4 | visits per year |
| | Peri-coital contraceptio | 4 | visits per year |
| Condom | Male Condom | 4 | visits per year |
| | Female Condom | 4 | visits per year |
| Other | LAM | 1 | counseling visit per user |
| | SDM (Standard Days) | 1 | counseling visit per user |
| | Vaginal barrier | 1 | years of protection |
| | Spermicides | 4 | visits per year of use |
| Emergency contraception | EC | 20 | visits per year of use |

Abbreviations: CYP, couple-years of protection; EC, emergency contraception; F, female; IUD, intrauterine device; LAM, Lactational Amenorrhea Method; LNG-IUS, levonorgestrel-releasing intrauterine system; M, male; SDM, Standard Days Method.

Magnani RJ, Ross J, Williamson J, Weinberger M. Can family planning statistics be used to track population-level outcomes? *Glob Health Sci Pract*. 2018;6(1). https://doi.org/10.9745/GHSP-D-17-00341

**SUPPLEMENT 3.** Calculation of Long-Acting Reversible Contraceptive Users From Service Visits and Commodities to Clients Data

Couple-years of protection (CYP) are designed to capture the impact of a given contraceptive method on providing protection against unintended pregnancy. While this measure can be very useful for program monitoring, it poses a challenge for estimating contraceptive coverage or users, specifically for long-acting methods.

For all modern methods, the CYP factor is applied to commodities and visits data in order to calculate the number of years of coverage provided. For short-acting methods, this coverage is fully realized in the year the services are provided. Therefore, total CYP in a year for short-acting methods can be used as a proxy for the number of women using each method at midyear. For long-acting methods, the CYP factor instead represents the number of years each individual receiving a method is protected into the future.

In a steady state, the CYP for long-acting methods can be used as a rough estimate of users. For example, it would assume that for each implant inserted, there are 2 to 3 women still using implants inserted in previous years. However, using the CYP can overestimate users during a period of rapid scale-up and underestimate users during a period of decline, as the entire impact of the method is counted in the year it is distributed, rather than spread over the years it is in use (see Figure A3.1)

**Figure A3.1: Illustration of CYP for Long-Acting Contraceptives in 3 Scenarios**

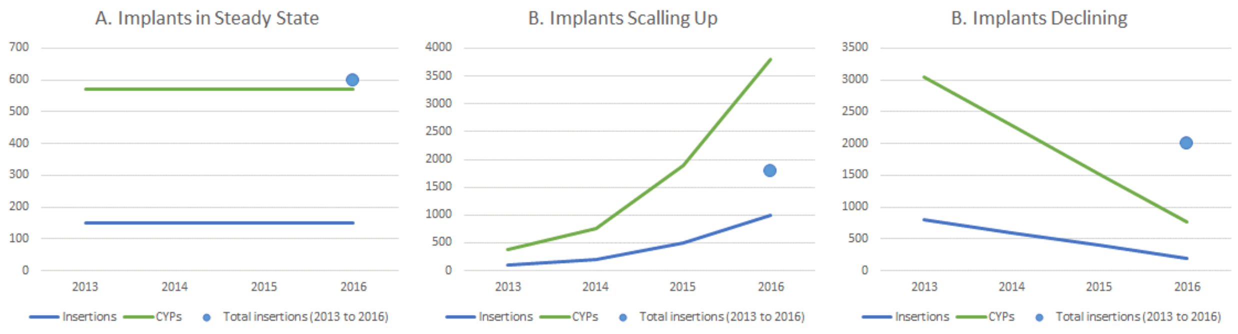


A second issue that arises when estimating users of long-acting methods is that while discontinuation of short-acting methods would generally be captured through service statistics (in that short-acting users are required to return regularly to receive their method, so stopping would result in fewer commodities and services being distributed), discontinuation of long-acting methods cannot be tracked based on commodity insertion or visit data alone.

In order to address these issues, a different approach was used to calculate "estimated users" of long-acting and reversible contraceptive (LARC) methods. The methodology used here is consistent with the methodology used by Marie Stopes International's Impact 2 model.[17]

Calculating the number of users of a LARC, such as intrauterine devices (IUDs) and implants, in a given year requires combining the following:

1. An estimate of the number of women provided with the method in that year (based on commodities/services data).
2. An estimate of the number of women who are still using a LARC provided in previous years.

**Calculating users provided with a method in the given year (item 1 above):** first-year discontinuation rates (see Table A3.1) are applied to the commodity/service data for LARCs (IUDs and implants). These are applied to the figures that have been adjusted to account for the missing private sector data as described in the body of this paper.

**Table A3.1. Method-Specific Continuation Rates**

| *Estimate of the percentage of women who received a method who are still using each year following receipt of the method* | | | | | | | | | | |
|---|---|---|---|---|---|---|---|---|---|---|
| | **Year** | | | | | | | | | |
| **Method** | 0 to 1 | 1 to 2 | 2 to 3 | 3 to 4 | 4 to 5 | 5 to 6 | 6 to 7 | 7 to 8 | 8 to 9 | 9 to 10 |
| Copper T 380A IUD | 92% | 77% | 65% | 54% | 46% | 38% | 32% | 27% | 23% | 19% |
| LNG IUS | 92% | 77% | 65% | 54% | 46% | 21% | 0% | | | |
| Implanon | 94% | 82% | 71% | 62% | 54% | 25% | 0% | | | |
| Sino-implant | 94% | 82% | 71% | 62% | 29% | 0% | | | | |
| Jadelle | 94% | 82% | 71% | 33% | 0% | | | | | |
| *Source: Marie Stopes International's Impact 2 model.* | | | | | | | | | | |

**Calculating users still using a method provided in a previous year (item 2 above):** For this calculation, the number of LARC insertions in past years is needed. Some of this data is available within a health management information system (HMIS), but depending on how long an HMIS has been in place, a full trend of historic data may not be available. In this case, historic services are estimated based on available data. This is done by projecting back a linear trend from the oldest year of data available; the trend is projected backward based on the duration of the given method (10 or 5 years for IUDs and 5, 4, or 3 years for implants). These estimates are needed to calculate the total users in each year; for example, IUD users in 2010 can include women who received their IUD as long ago as 2000. An illustration of this process is shown in Table A3.2, for a case where, in 2009, a total of 5,000 10-year IUDs were distributed. No data is

available for pre-2009, so historic IUD insertions must be estimated. To do this, a linear trend is created going back 10 years.

**Table A3.2. Estimating Historic IUD Services**

| | Estimated Services by Year | | | | | | | | | | Documented Services |
|---|---|---|---|---|---|---|---|---|---|---|---|
| | **1999** | **2000** | **2001** | **2002** | **2003** | **2004** | **2005** | **2006** | **2007** | **2008** | **2009** |
| IUDs distributed | 0 | 500 | 1000 | 1500 | 2000 | 2500 | 3000 | 3500 | 4000 | 4500 | 5000 |

Once a full trend of service provision data is available, the cumulative number of users still using a method received in past years can then be calculated. This is done by applying the method-specific continuation rates shown previously in Table A3.1. Essentially, a cohort of users is traced out for each set of insertions in past years, then, by summing across these cohorts, the total number of users still using a LARC received in past years can be calculated. Adding in the number of women using a LARC based on those who received a method in the current year (item 1 from above), the total number of users can be estimated. An illustration of this is shown in Table A3.3, where it is estimated that 16,255 women are using an IUD in 2009.

**Table A3.3. Estimating Total Number of Users From Trends in IUD Insertions**

| | | Year IUD in Use | | | | | | | | | |
|---|---|---|---|---|---|---|---|---|---|---|---|
| | | 2000 | 2001 | 2002 | 2003 | 2004 | 2005 | 2006 | 2007 | 2008 | 2009 |
| Year IUD Provided | 2000 | 460 | 385 | 325 | 270 | 230 | 190 | 160 | 135 | 115 | 95 |
| | 2001 | | 920 | 770 | 650 | 540 | 460 | 380 | 320 | 270 | 230 |
| | 2002 | | | 1380 | 1155 | 975 | 810 | 690 | 570 | 480 | 405 |
| | 2003 | | | | 1840 | 1540 | 1300 | 1080 | 920 | 760 | 640 |
| | 2004 | | | | | 2300 | 1925 | 1625 | 1350 | 1150 | 950 |
| | 2005 | | | | | | 2760 | 2310 | 1950 | 1620 | 1380 |
| | 2006 | | | | | | | 3220 | 2695 | 2275 | 1890 |
| | 2007 | | | | | | | | 3680 | 3080 | 2600 |
| | 2008 | | | | | | | | | 4140 | 3465 |
| | 2009 | | | | | | | | | | 4600 |
| **Total IUD Users** | | **460** | **1,305** | **2,475** | **3,915** | **5,585** | **7,445** | **9,465** | **11,620** | **13,890** | **16,255** |

This methodology allows for more accurate estimates of the total number of LARC users, especially in cases where there is rapid scale-up or declines in the number of insertions. However, this methodology is not without limitations. Projecting a simple linear trend backward based on method duration provides a means of estimating some level of historic use, but it does not fully represent historic use. This methodology may underestimate continuing users in countries with a long history of method provision or relatively consistent levels of provision in years prior to available data. Alternative approaches are being explored that can

better account for estimating historic trends. However, even with these limitations, we feel that this approach is still preferable and more accurate than simply using the CYP factor for LARCs.

Finally, it should be noted that we did not apply this methodology to sterilization numbers, as such a calculation is more complex and because in most countries, overall sterilization numbers are fairly low and/or relatively static (in which case the CYP factor is a decent approximation). However, there are a few countries where the use of the CYP factor for sterilizations does not provide an accurate estimate, and work is underway to develop an approach comparable to that described for LARCs to apply in these cases.

Magnani RJ, Ross J, Williamson J, Weinberger M. Can family planning statistics be used to track population-level outcomes? *Glob Health Sci Pract*. 2018;6(1). https://doi.org/10.9745/GHSP-D-17-00341

**SUPPLEMENT 4.** Formulae for Computing the Components of Mean Square Error

The calculations of the components of mean square error (MSE) were performed as described below.

*Variance* was measured as the squared deviations between the annual estimated modern use (EMU) rate and the value for the corresponding year on the EMU least-squares linear trend line; the sum of these was divided by n, in which n = the number of observations. That is,

$$VAR_{EMU} = \sum(EMU_{year\ i} – EMU\ Trend\ Line_{year\ i})^2 / n$$

Where:

n = the number of years for which service statistics–based estimates are available.

Note that the formula we used to calculate variance differs from more commonly used versions in that it divides by the number of years rather than by the mean EMU. The formula we used gives the average of the squared deviations across the years, rather than the single mean for all the original observations. The use of the former is more appropriate when dealing with time series data.

Next, because countries with higher modern contraceptive prevalence rates (mCPRs) have a potential for greater variability in EMU rates than countries with lower mCPRs do, we used the relative variance (RelVar), which adjusts variance according to the overall level (mean) of EMU.

The RelVar was calculated as:

$$RelVar_{EMU} = VAR_{EMU} / Mean_{EMU}$$

Where:

$Mean_{EMU}$ = the average of EMU values included in the variance calculation.

*Level bias* was measured as the mean difference (absolute values) between annual EMU trend line values and the Family Planning Estimation Tool (FPET) estimates of mCPR. That is,

$$Bias_{Level} = ((\sum ABS(EMU\ Trend\ Line_{year\ i} – FPET_{year\ i}) / n) / Mean\ FPET)$$

Where:

n = number of years for which both survey-based and service statistics–based estimates are available, and

Mean FPET = $\sum$mCPR(FPET $_{\text{year i}}$) / n

As with estimates of variance, the level bias estimates were adjusted to account for the fact that countries with higher mCPRs had potential for higher levels of bias measured in absolute terms. This potential distortion was mitigated by dividing the estimates of absolute level bias by the mean value of the FPET-based mCPR estimates during the reference period for each country (as shown in the formula above).

*Trend bias* was measured as the absolute value of the difference between the 2 slopes: 1 for the service statistics–based EMU rates and 1 for the survey-based FPET estimates of the mCPR. That is,

Slope Bias = ABS (Slope EMU Trend Line – Slope Survey FPET Trend Line)

Magnani RJ, Ross J, Williamson J, Weinberger M. Can family planning statistics be used to track population-level outcomes? *Glob Health Sci Pract*. 2018;6(1). https://doi.org/10.9745/GHSP-D-17-00341

**SUPPLEMENT 5.** Country Calculations of MSE

| Country | Commodities to Clients | | | | Service Visits | | | | Current Users | | | |
|---|---|---|---|---|---|---|---|---|---|---|---|---|
| | MSE | Variance | Level Bias | Slope Bias | MSE | Variance | Level Bias | Slope Bias | RMSE | Variance | Level Bias | Slope Bias |
| 1 | 0.1310 | 0.0102 | 0.9073 | 0.0189 | -- | -- | -- | -- | -- | -- | -- | -- |
| 2 | 0.1625 | 0.0118 | 0.2704 | 0.0065 | -- | -- | -- | -- | -- | -- | -- | -- |
| 3 | 0.1625 | 1.6820 | 0.0355 | 0.0299 | -- | -- | -- | -- | -- | -- | -- | -- |
| 4 | 0.1488 | 0.0003 | 0.4031 | 0.0130 | -- | -- | -- | -- | -- | -- | -- | -- |
| 5 | 0.0107 | 0.0001 | 0.5511 | 0.0003 | -- | -- | -- | -- | -- | -- | -- | -- |
| 6 | 0.0964 | 0.0010 | 0.5775 | 0.0071 | -- | -- | -- | -- | -- | -- | -- | -- |
| 7 | 0.1625 | 0.0014 | 0.1562 | 0.0198 | -- | -- | -- | -- | -- | -- | -- | -- |
| 8 | 0.0433 | 0.0056 | 0.2169 | 0.0421 | -- | -- | -- | -- | -- | -- | -- | -- |
| 9 | 0.1474 | 0.0004 | 0.3114 | 0.0080 | -- | -- | -- | -- | -- | -- | -- | -- |
| 10 | 0.0695 | 0.0031 | 0.2213 | 0.0366 | -- | -- | -- | -- | -- | -- | -- | -- |
| 11 | 0.0344 | 0.0004 | 0.1749 | 0.0114 | -- | -- | -- | -- | 0.0184 | 0.0013 | 0.0355 | 0.0042 |
| 12 | 0.0550 | 0.0010 | 0.3348 | 0.0044 | -- | -- | -- | -- | 11.4994 | 0.2394 | 72.3301 | 0.3369 |
| 13 | 0.3116 | 0.2938 | 0.3678 | 0.0232 | -- | -- | -- | -- | 0.3304 | 0.0076 | 0.5904 | 0.2691 |
| 14 | 0.0404 | 0.0033 | 0.2748 | 0.0161 | -- | -- | -- | -- | 0.2317 | 0.0288 | 1.9125 | 0.0061 |
| 15 | 0.0864 | 0.0002 | 0.3103 | 0.0007 | -- | -- | -- | -- | 0.1574 | 0.0006 | 0.5644 | 0.0079 |
| 16 | 0.1174 | 0.0017 | 0.2265 | 0.0418 | 0.7565 | 0.0037 | 1.7362 | 0.0663 | 0.2811 | 0.0008 | 0.6478 | 0.0156 |
| 17 | 0.1598 | 0.0001 | 0.4430 | 0.0195 | 0.1636 | 0.0000 | 0.6015 | 0.0158 | 0.2115 | 0.0001 | 0.5624 | 0.0059 |
| 18 | 0.0543 | 0.0040 | 0.4578 | 0.0018 | 0.0307 | 0.0033 | 0.2704 | 0.0064 | -- | -- | -- | -- |
| 19 | 0.0466 | 0.0001 | 0.5757 | 0.0013 | 0.0232 | 0.0001 | 0.2952 | 0.0011 | -- | -- | -- | -- |
| 20 | 0.0463 | 0.0001 | 0.1031 | 0.0034 | 0.0727 | 0.0000 | 0.1688 | 0.0151 | -- | -- | -- | -- |
| 21 | 0.2294 | 0.0025 | 0.5002 | 0.0023 | 0.3093 | 0.0000 | 0.6773 | 0.0073 | -- | -- | -- | -- |
| 22 | -- | -- | -- | -- | 0.1149 | 0.0003 | 0.2371 | 0.0049 | 0.0565 | 0.0011 | 0.0991 | 0.0129 |

Abbreviation: MSE, mean square error.
